# High-fat diet decreases activity of the oxidative phosphorylation complexes and causes nonalcoholic steatohepatitis in mice

**DOI:** 10.1242/dmm.016766

**Published:** 2014-09-26

**Authors:** Inmaculada García-Ruiz, Pablo Solís-Muñoz, Daniel Fernández-Moreira, Montserrat Grau, Francisco Colina, Teresa Muñoz-Yagüe, José A. Solís-Herruzo

**Affiliations:** 1 Research Center, Laboratory of Gastroenterology and Hepatology, University Hospital ‘12 de Octubre’, Complutense University, 28041-Madrid, Spain.; 2 Institute of Liver Studies, King’s College Hospital, London, SE5 9RS, UK.; 3 Department of Bromatology and Food Hygiene, Military Center of Veterinary of Defense, 28024-Madrid, Spain.; 4 Department of Pathology, University Hospital ‘12 de Octubre’, Complutense University, 28041-Madrid, Spain.

**Keywords:** Mitochondrial respiratory chain, Nonalcoholic steatohepatitis, NADPH oxidase, Oxidative phosphorylation, Proteomic, Nitro-oxidative stress

## Abstract

Nonalcoholic fatty liver disease (NAFLD) is the most frequent histological finding in individuals with abnormal liver-function tests in the Western countries. In previous studies, we have shown that oxidative phosphorylation (OXPHOS) is decreased in individuals with NAFLD, but the cause of this mitochondrial dysfunction remains uncertain. The aims of this study were to determine whether feeding mice a high-fat diet (HFD) induces any change in the activity of OXPHOS, and to investigate the mechanisms involved in the pathogenesis of this defect. To that end, 30 mice were distributed between five groups: control mice fed a standard diet, and mice on a HFD and treated with saline solution, melatonin (an antioxidant), MnTBAP (a superoxide dismutase analog) or uric acid (a scavenger of peroxynitrite) for 28 weeks intraperitoneously. In the liver of these mice, we studied histology, activity and assembly of OXPHOS complexes, levels of subunits of these complexes, gene expression of these subunits, oxidative and nitrosative stress, and oxidative DNA damage. In HFD-fed mice, we found nonalcoholic steatohepatitis, increased gene expression of *TNFα*, *IFNγ*, *MCP-1*, caspase-3, *TGFβ1* and collagen α1(I), and increased levels of 3-tyrosine nitrated proteins. The activity and assembly of all OXPHOS complexes was decreased to about 50–60%. The amount of all studied OXPHOS subunits was markedly decreased, particularly the mitochondrial-DNA-encoded subunits. Gene expression of mitochondrial-DNA-encoded subunits was decreased to about 60% of control. There was oxidative damage to mitochondrial DNA but not to genomic DNA. Treatment of HFD-fed mice with melatonin, MnTBAP or uric acid prevented all changes observed in untreated HFD-fed mice. We conclude that a HFD decreased OXPHOS enzymatic activity owing to a decreased amount of fully assembled complexes caused by a reduced synthesis of their subunits. Antioxidants and antiperoxynitrites prevented all of these changes, suggesting that nitro-oxidative stress played a key role in the pathogenesis of these alterations. Treatment with these agents might prevent the development of NAFLD in humans.

## INTRODUCTION

Nonalcoholic fatty liver disease (NAFLD) represents a spectrum of liver diseases that occur in individuals who do not consume a significant amount of alcohol, extending from pure fatty liver through nonalcoholic steatohepatitis (NASH) to cirrhosis and hepatocarcinoma (Matteoni et al., 1999). NAFLD has emerged as a worldwide common problem that represents the most frequent histological finding in individuals with abnormal liver tests in the Western countries (McCullough, 2005).

Although the pathogenesis of NAFLD remains undefined, the so-called ‘two hits’ model of pathogenesis has been proposed (Day and James, 1998). Whereas the ‘first hit’ involves the accumulation of fat in the liver, the ‘second hit’ involves oxidative stress, resulting in inflammation, stellate cell activation and fibrogenesis (Chitturi and Farrell, 2001). Mitochondrial dysfunction might play a crucial role in the induction of both ‘hits’, because mitochondria are involved in the β-oxidation of free fatty acids (FFAs), and are the most important source of reactive oxygen species (ROS) (Fromenty et al., 2004). In previous studies, we have shown that oxidative phosphorylation (OXPHOS) (supplementary material Fig. S1) is defective in individuals with NASH (Pérez-Carreras et al., 2003) and in *ob/ob* mice with NAFLD (García-Ruiz et al., 2006). In *ob/ob* mice, we found evidence indicating that inhibition of OXPHOS was caused by a reduced amount of fully assembled complexes that was due to decreased synthesis and increased degradation of its subunits by the nitro-oxidative stress. We proposed that abdominal obesity might contribute to increase the tumor necrosis factor-α (TNFα) and FFA levels in the liver and that these factors could induce oxidative and nitrosative stress. Indeed, TNFα increases superoxide anion formation (Sánchez-Alcázar et al., 2000) and upregulates mitochondrial inducible nitric oxide synthase (*iNOS*) gene expression, resulting in the formation of nitric oxide (García-Ruiz et al., 2006). Likewise, saturated FFAs increase superoxide anion production (Lambertucci et al., 2012). The combination of both superoxide and nitric oxide leads to the formation of peroxynitrite (Pryor and Squadrito, 1995), which can nitrate tyrosine residues within mitochondrial proteins and cause degradation of mitochondrial complexes and the loss of OXPHOS activity (Murray et al., 2003; García-Ruiz et al., 2010). This is the last step in a vicious circle that could progressively worsen the mitochondrial function. Consistent with this hypothesis is the fact that treatment of obese mice with anti-TNFα or uric acid (García-Ruiz et al., 2006), a scavenger of peroxynitrite, or with melatonin (MLT), a broad-spectrum antioxidant that is capable of neutralizing peroxynitrite and its derivatives (Soung et al., 2004; Cuzzocrea et al., 1997), prevented mitochondrial dysfunction and NAFLD liver lesions found in *ob/ob* mice (Solís-Muñoz et al., 2011). However, *ob/ob* mice are not the ideal model to study NAFLD because they do not develop inflammation and fibrosis and do not spontaneously progress from steatosis to NASH. By contrast, mice fed a high-fat diet (HFD) are considered a valuable tool for investigating NAFLD (Ito et al., 2007).

**TRANSLATIONAL IMPACT**

**Clinical issue**

Nonalcoholic fatty liver disease (NAFLD) is a worldwide problem that represents the most frequent histological finding in individuals with abnormal liver tests in the Western countries. NAFLD pathogenesis remains undefined but recent studies have found that oxidative phosphorylation (OXPHOS) is decreased in individuals with this disease. The OXPHOS system is the metabolic pathway by which mitochondria use energy released by oxidation of nutrients to produce ATP, which supplies energy to cell metabolism. Mitochondria are involved in the oxidation of fatty acids and are important sources of reactive oxygen species (ROS). Therefore, defective OXPHOS might contribute to the accumulation of fat in the liver and cause oxidative stress, resulting in inflammation and progression of the disease to steatohepatitis, cirrhosis and eventually hepatocarcinoma. The cause of this dysfunction is unknown. The aim of the present study was to determine whether a high-fat diet (HFD) could decrease OXPHOS activity and to elucidate the mechanisms involved in the pathogenesis of the OXPHOS defect.

**Results**

In HFD-fed mice, the authors found hepatic lesions of nonalcoholic steatohepatitis and a marked decrease in OXPHOS activity. This decrease was due to a reduction in the amount of fully assembled OXPHOS enzyme complexes and to a marked decrease in the amount of complex subunits, particularly those encoded by mitochondrial DNA. The authors found that a HFD induced oxidative damage to the mitochondrial DNA. Liver tissue and liver proteins, including OXPHOS proteins, were 3-tyrosine nitrated. The use of antioxidants, such as melatonin, or antiperoxynitrites, such as uric acid, prevented all the changes that were observed in untreated HFD-fed mice, including lesions of nonalcoholic steatohepatitis.

**Implications and future directions**

This study demonstrates that a HFD reduces OXPHOS enzymatic activity by decreasing the amount of fully assembled complexes, and that this defect is caused by reduced synthesis of their subunits. Because antioxidants and antiperoxynitrites prevented all these changes, treatment with these agents might be useful in preventing the development of NAFLD in humans. The mechanisms by which HFD induces nitro-oxidative stress remains unclear, but NADPH oxidase might be involved. Therefore, further studies should better investigate these mechanisms and, in particular, address whether: (1) fatty acids are involved in the pathogenesis of this effect of HFD, (2) increased degradation of OXPHOS subunits might contribute to reduced OXPHOS activity, (3) NADPH oxidase is responsible for the HFD-induced nitro-oxidative stress, (4) inhibition of NADPH oxidase prevents OXPHOS dysfunction and nonalcoholic steatohepatitis, and (5) fatty acids are able to activate this oxidase.

The aims of the present study were to determine: (a) whether mice on a HFD develop changes in the OXPHOS enzyme activity and (b) to establish mechanisms involved in the pathogenesis of this defect.

## RESULTS

### Effects of a HFD with or without MnTBAP, MLT or uric acid

As Table 1 shows, the body weight gain over the 28 weeks of the experiment was significantly higher in mice on a HFD than in animals fed the standard diet. This increase was associated with increased levels of hepatic triglyceride (*r*, 0.477; *P*<0.01) and hepatic FFAs (*r*, 0.372; *P*<0.05). In addition to obesity, mice on a HFD developed other features of the metabolic syndrome, including hyperglycemia, hypertriglyceridemia, increased plasma FFAs and low levels of plasma adiponectin. Because nitro-oxidative stress could play a major role in the pathogenesis of this disease, we treated HFD-fed animals with manganese [III] tetrakis (5,10,15,20 benzoic acid) porphyrin (MnTBAP; a superoxide dismutase mimic), MLT (an antioxidant) or uric acid (a scavenger of peroxynitrite) for 28 weeks. Mice treated with these agents also increased the body weight significantly. However, hepatic triglyceride and FFA concentrations were significantly lower in these mice than in untreated HFD-fed mice. Serum levels of aspartate aminotransferase (AST) and alanine transaminase (ALT) were markedly increased in HFD-fed mice (*P*<0.001), but treatment with MnTBAP, MLT or uric acid decreased these levels significantly.

**Table 1.**
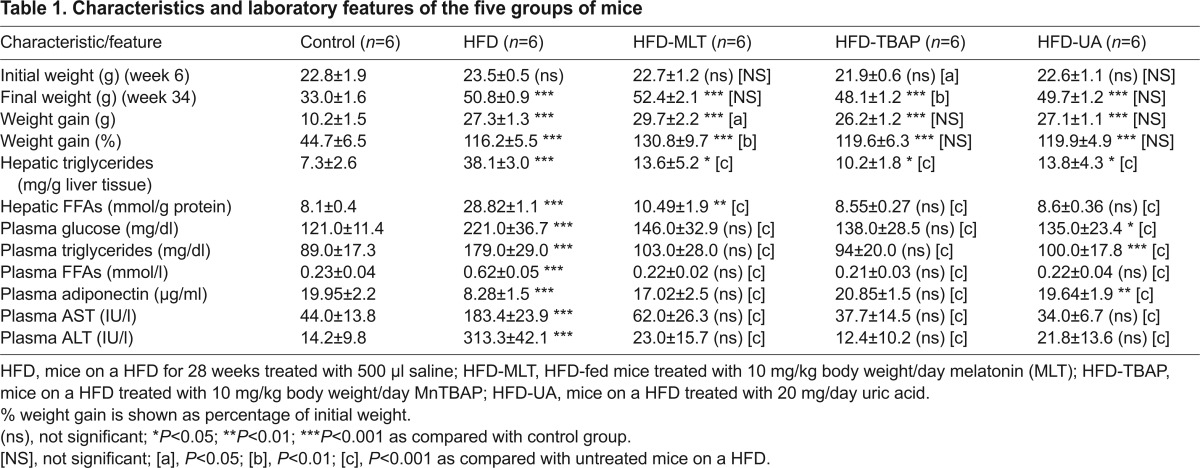
Characteristics and laboratory features of the five groups of mice

### HFD induced non-alcoholic steatohepatitis

**Fig. 1.**
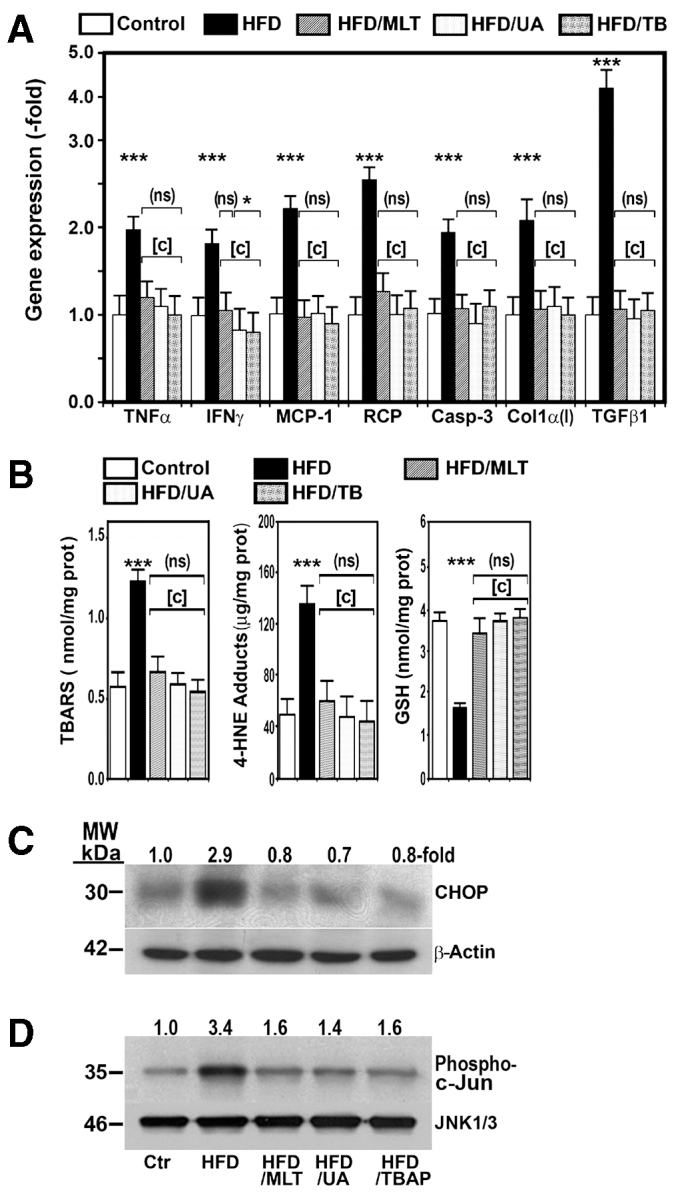
**A HFD increased gene expression of proinflammatory, apoptotic and fibrogenic factors, and induced oxidative stress and endoplasmic reticulum stress in mouse liver.** Ctr, control mice; HFD, mice on a HFD treated with saline i.p. for 28 weeks; HFD/MLT, HFD mice treated with melatonin; HDF/UA, HFD mice treated with uric acid; HFD/TBAP or HFD/TB, HFD mice treated with MnTBAP. Values are shown as mean ± s.d. (ns), not significant; ***P<0.001 versus control mice. [c], *P*<0.001 versus mice on a HFD. (A) Gene expression of TNFα, IFNγ, MCP-1, reactive C protein (RCP), caspase-3 and collagen α1(I) was measured by RT-PCR. (B) Thiobarbituric acid reactive substances (TBARS), 4-hydroxynonenal-protein (4-HNE) adducts and reduced glutathione (GSH) were measured in the liver as described in the Materials and Methods. (C) Mitochondrial proteins were isolated from control mice and mice on a HFD treated as indicated above and analyzed by western blotting. Membrane was probed with specific antibody against C/EBP homologous protein (CHOP) and β-actin. Results are expressed as fold over the control level. (D) c-Jun N-terminal kinase (JNK) activity was measured as described in the Materials and Methods. JNK was precipitated by adding c-Jun fusion beads to hepatic lysates. After centrifugation, pellets were suspended in kinase assay buffer in the presence of ATP. Supernatants were loaded on an SDS-PAGE. Western blot analysis was performed using rabbit anti-phospho-c-Jun-specific antibody. Levels of JNK were used to demonstrate equal loading. These results are representative of two separate experiments.

**Fig. 2.**
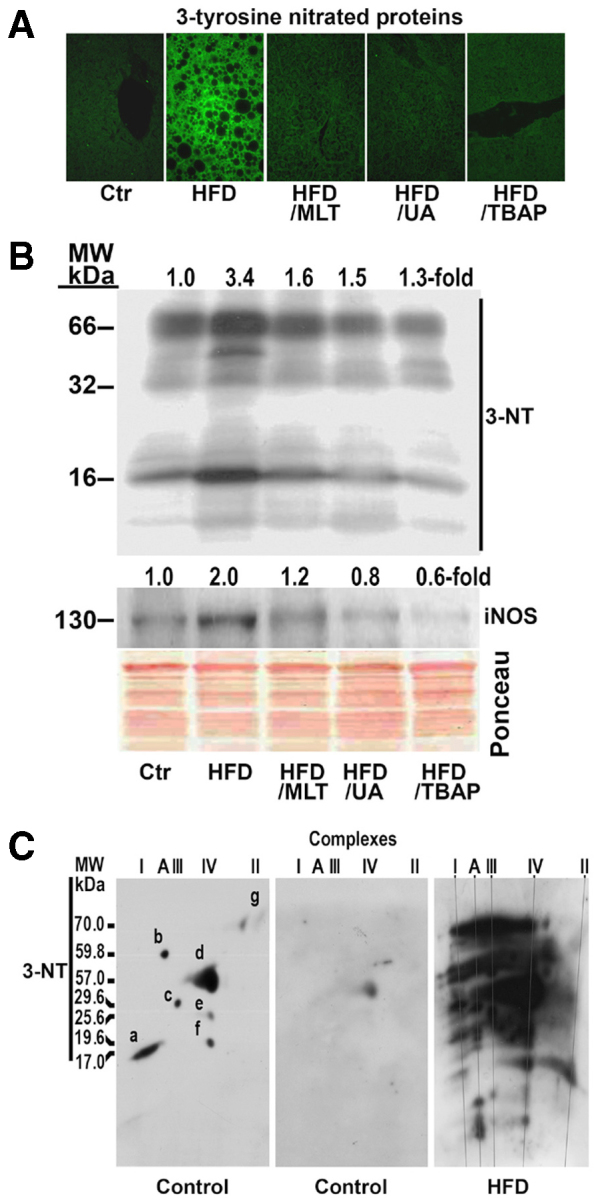
**A HFD induced nitrosative stress in mouse liver.** Mice were treated as indicated in Fig. 1. (A) Formalin-fixed, paraffin-embedded sections were deparaffinized and then incubated with anti-3-nitrotyrosine antibody followed by incubation with anti-rabbit IgG FITC-conjugated secondary antibody. Ctr, tyrosine nitrated proteins in the liver of a control mouse. (B) Tyrosine nitrated mitochondrial proteins. Proteins were extracted from hepatic mitochondria and analyzed by western blotting. Membranes were probed with specific antibody against 3-nitrotyrosine (3-NT), and inducible oxide nitric synthase (iNOS). Ponceau S staining shows equal loading of protein per line. MW, molecular weight. (C) 3-tyrosine nitrated proteins in liver mitochondria of a control mouse and a mouse on a HFD. Membrane of a control mouse was first probed with antibody against NDUFB6 (a), ATP5A1 (b), UQCRFS1 (c), MTCO1 (d), MTCO2 (e), COX4 (f) and SDHA (g), and, after removing these antibodies, was probed with specific antibody against 3-NT. Complex A, ATP synthase.

As compared with control mice, the liver of HFD-fed mice revealed severe steatosis, ballooning degeneration, Mallory and eosinophilic bodies, scattered mixed neutrophilic-lymphocytic inflammatory foci, and increased perisinusoidal fibrosis (supplementary material Fig. S2). These histological results were supported by the analysis of hepatic triglyceride levels (Table 1), and gene expression of four inflammatory markers [TNFα, monocyte chemoattractant protein-1 (MCP-1), IFNγ and reactive C protein], a marker of apoptotic death (caspase-3) and fibrogenesis markers [TGFβ1 and collagen α1(I)], levels of all of which were significantly increased in the liver of HFD-fed mice (Fig. 1A). In these livers, we also found phosphorylation of c-jun N-terminal kinase (JNK), an important mediator of cell death, and evidence of oxidative stress, endoplasmic reticulum stress and nitrosative stress. Thus, mice on a HFD had a marked increase in thiobarbituric acid reactive substances (TBARS) and 4-hydroxynonenal, and a decrease in reduced glutathione (GSH) (Fig. 1B). Likewise, protein expression of C/EBP homologous protein (CHOP) (Fig. 1C), a marker of endoplasmic reticulum stress (Haataja et al., 2008), and iNOS and 3-tyrosine nitrated proteins (Fig. 2) were markedly increased in these mice. No such changes were observed in HFD mice treated with uric acid, MnTBAP or MLT (Figs 1, 2).

### Activity of the OXPHOS enzyme complexes was decreased in mice on a HFD

**Fig. 3.**
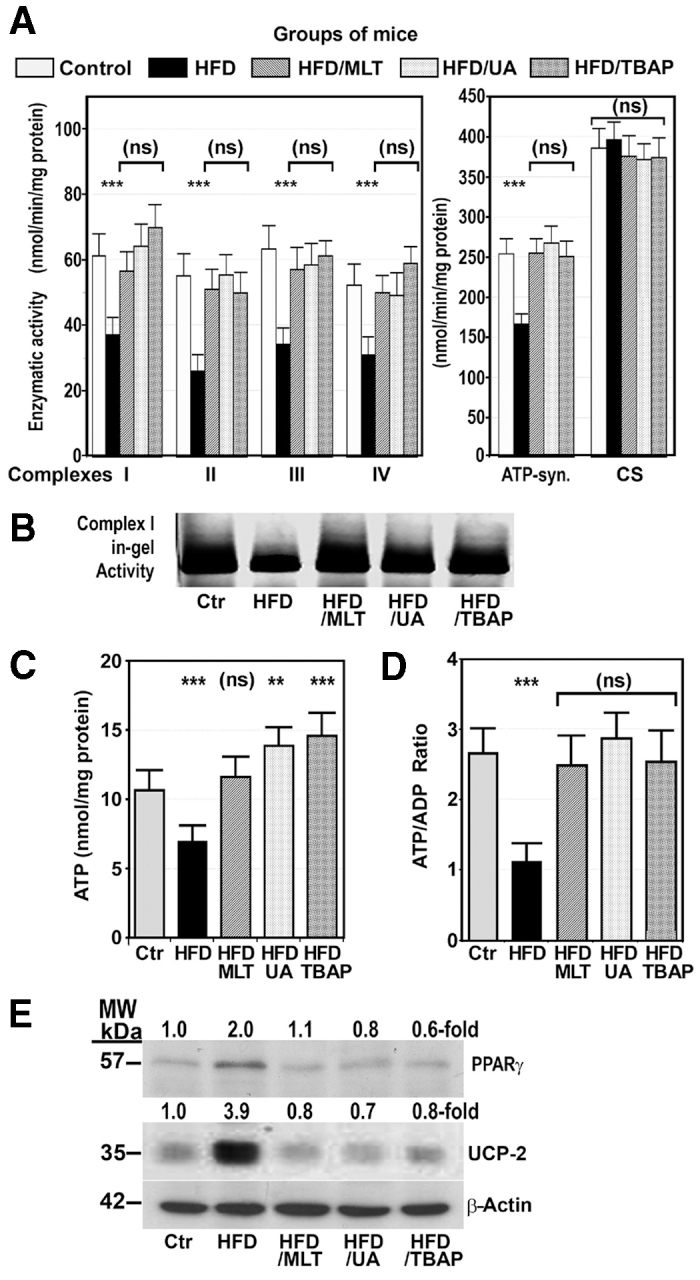
**A HFD decreased activity of the OXPHOS complexes, reduced hepatic ATP content, and increased protein expression of UCP-2 and PPARγ.** Mice were treated as described in Fig. 1. Values are shown as mean ± s.d. (ns), not significant; **P<0.01; ***P<0.001 versus control mice. ATP-syn., ATP synthase; CS, citrate synthase. (A) Activity of the OXPHOS enzyme complexes and CS was measured as indicated in the Materials and Methods and expressed as nmol of substrate used per minute per mg protein, and is referred to as a percentage of the specific activity of CS. (B) Complex-I in-gel activity was displayed as described in the Materials and Methods. (C,D) ATP content (C) and ATP:ADP ratio (D) in the liver of mice treated as indicated. (E) Western blot showing hepatic protein expression of UCP-2, PPARγ and β-actin in the same groups of mice as above. MW, molecular weight.

We measured the enzymatic activity of the OXPHOS complexes in the liver of both control and HFD-fed mice. The activity of complex I was decreased from 62.7±8.8 nmol/min/mg protein in control mice to 37.5±7.2 nmol/min/mg protein (*P*<0.001; 59.8±11.4%) in mice on a HFD (Fig. 3A). To correct for mitochondrial volume, all OXPHOS enzyme activities were normalized to the activity of citrate synthase (CS). This decrease was also observed by measuring in-gel complex I activity (Fig. 3B). In HFD-fed mice treated with MLT, MnTBAP or uric acid, activity of complex I was increased to the control levels.

In HFD-fed mice, the activity of complex II was significantly reduced to 46.7±11.1% (*P*<0.001) of control activity. This decrease was not observed in HFD-fed mice treated with MLT, MnTBAP or uric acid (Fig. 3A).

In HFD-fed mice, the activity of complex III was reduced to 54.1±8.1% (*P*<0.001) of control values (Fig. 3A), but treatment of HFD-fed mice with MLT, MnTBAP or uric acid avoided this decrease.

Measurement of the activity of complex IV in the liver mitochondria of HFD mice showed that it was significantly decreased to 59.2±6.9% (*P*<0.001) of the control activity. This activity was normal in HFD-mice treated with MLT, MnTBAP or uric acid (Fig. 3A).

The activity of ATP synthase was significantly reduced to 67.3±13.1% (*P*<0.001) of the control activity in HFD-fed mice but, in mice treated with MLT, MNTBAP or uric acid, this activity was not different from the activity in control mice (Fig. 3A).

Specific activities of CS were 384.3±85.6 nmol/min/mg protein for control mice, 396.9±81.2 nmol/min/mg protein for HFD-fed mice, 371.7±89.1 nmol/min/mg protein for mice on HFD treated with MLT, 369.0±49.7 nmol/min/mg protein for mice treated with MnTBAP, and 367.7±68.4 nmol/min/mg protein for mice treated with uric acid, indicating equal mitochondria volume in all groups of mice (Fig. 3A).

### ATP content and ATP:ADP ratio were decreased in mice on a HFD

Because the final product of OXPHOS is ATP, we measured the ATP content and the ATP:ADP ratio in the liver of control and HFD-fed mice with or without treatment with MLT, MnTBAP or uric acid. As Fig. 3C shows, a HFD decreased hepatic ATP from 10.7±1.4 nmol/mg protein to 7.0±1.3 nmol/mg protein (*P*<0.001). However, treatment of HFD-fed mice with MLT, MnTBAP or uric acid increased hepatic ATP contents over the control levels. Likewise, the ATP:ADP ratio was significantly decreased in mice on a HFD but returned to the control levels in obese mice treated with MLT, MnTBAP or uric acid (Fig. 3D).

Finally, uncoupling protein-2 (UCP-2) – a protein that promotes translocation of protons from the intermembrane space to the mitochondrial matrix, reducing the proton gradient before it can be used to provide the energy for OXPHOS – and peroxisome proliferator-activated receptor-γ (PPARγ) – a transcription factor that upregulates *UCP-2* gene expression – were markedly increased in HFD-fed mice. This increase was not found in HFD mice treated with MLT, MnTBAP or uric acid for 28 weeks (Fig. 3E).

### Fully assembled OXPHOS complexes are decreased in the liver of HFD-fed mice

**Fig. 4.**
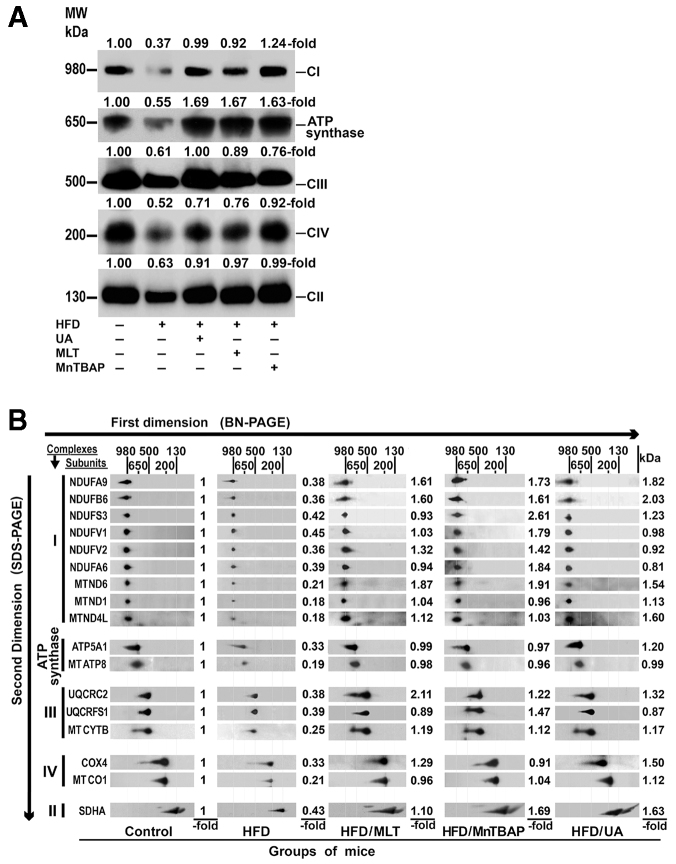
**A HFD reduced fully assembled OXPHOS complexes and the amount of complex subunits.** (A) BN-PAGE analysis of OXPHOS complexes in mice treated as indicated in Fig. 1. Western blot analysis of mitochondrial proteins was performed using antibody against subunits of complex I, II, III and IV, and ATP synthase. MW, molecular weight. (B) Mitochondrial proteins extracted from the same groups of mice as above were separated in the first dimension using BN-PAGE and in the second dimension using SDS-PAGE. The presence of individual subunits of these complexes was identified by immunoblotting using appropriated antibodies. -fold, amount of subunit in HFD-fed mice divided by the amount of the same subunit in control mice.

The first-dimension BN-PAGE system illustrates that the abundance of fully assembled complexes was markedly diminished in HFD-fed mice as compared with control mice (Fig. 4A), which concurs with the decreased OXPHOS-complex activity found in these obese mice. However, treatment of mice on a HFD with MLT, MnTBAP or uric acid for 28 weeks normalized the amount of fully assembled complexes in mitochondrial preparations (Fig. 4A).

### The amount of OXPHOS subunits was markedly reduced in HFD-fed mice

To study how mitochondrial complex subunits were affected by the HFD, complexes were resolved by second-dimension SDS-PAGE and nuclear DNA (nDNA)- and mitochondrial DNA (mtDNA)-encoded subunits were detected using specific antibodies. As Fig. 4B shows, the most striking finding was a fall in the amount of all studied OXPHOS subunits in HFD-fed mice. This reduction was particularly marked in mtDNA-encoded subunits. Thus, although the amount of nDNA-encoded subunits was decreased to 38.4±3.8% of control values in HFD mice, mtDNA-encoded subunits were reduced to only 20.3±2.6% of the amount found in control mice (*P*<0.0001). In none of the subunits tested was an accumulation of low-molecular-weight subcomplexes recognized. Treatment of these obese mice with MLT, MnTBAP or uric acid increased the protein content of all subunits, frequently over the control levels (Fig. 4B).

### Mitochondrial gene transcription was decreased in the liver of HFD-fed mice

**Fig. 5.**
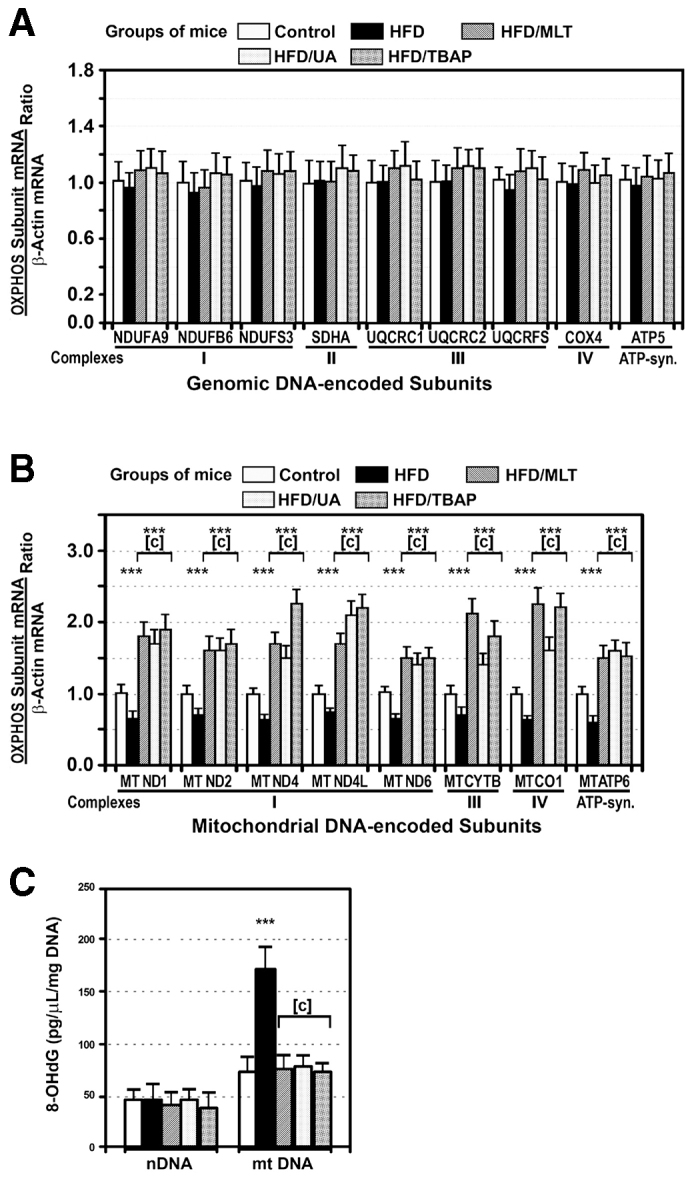
**A HFD decreased gene expression of mtDNA-encoded subunits of the OXPHOS complexes and increased oxidation of mtDNA.** Gene expression of representative nuclear-encoded (A) and mtDNA-encoded (B) OXPHOS subunits was analyzed by quantitative real-time PCR in mice treated as described in Fig. 1. Gene expression was expressed as subunit:β-actin mRNA ratio. (C) 8-OHdG content was measured in nDNA and mtDNA of the same mice described in A and B. ***P<0.001 as compared with control mice. [c], *P*<0.001 as compared with mice on a HFD treated with saline.

To determine gene expression of subunits of the OXPHOS complexes, we examined the steady-state levels of nDNA- and mtDNA-encoded mRNA in the liver of control and HFD-fed mice. This study revealed that gene expression of nDNA-encoded subunits was similar in both groups of mice (Fig. 5A), whereas expression of mtDNA-encoded subunits was reduced to 54.3±6.0% control levels in the obese animals. Treatment of mice on HFD with melatonin, MnTBAP or uric acid increased gene expression of mtDNA-encoded subunits over control levels (Fig. 5B).

### HFD caused oxidative damage to mtDNA

The 8-hydroxy-2′-deoxyguanosine (8-OHdG) content in nDNA was identical in HFD-fed mice and control mice (Fig. 5C). However, compared with the content of 8-OHdG in nDNA, this marker for oxidative DNA damage was significantly increased in the mtDNA isolated from all groups of mice, but it was particularly marked in mtDNA from mice on a HFD (Fig. 5C). Levels of 8-OHdG in mtDNA decreased to control levels in HFD-fed mice treated with MLT, MnTBAP or uric acid.

### NADPH oxidase gene expression and activity were increased in HFD-fed mice

**Fig. 6.**
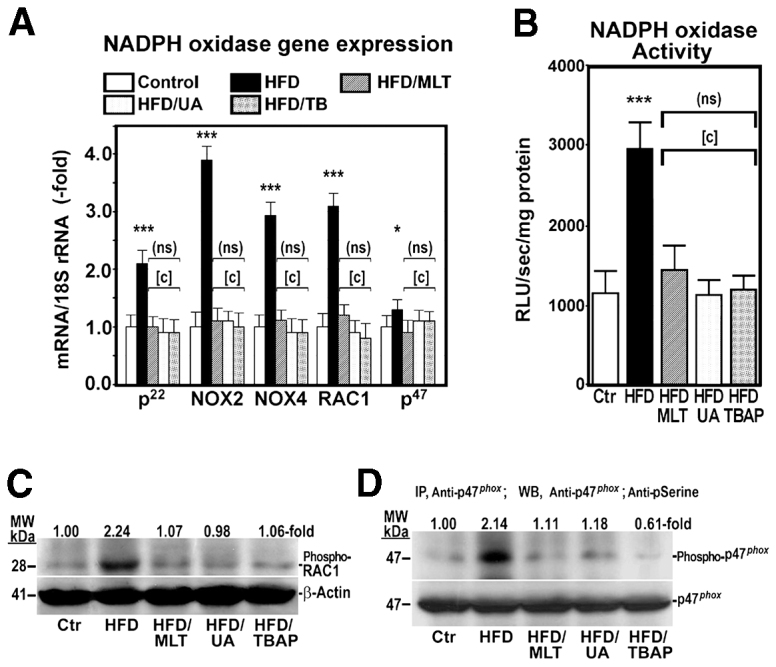
**A HFD upregulates gene expression and activity of NADPHox in mice.** NADPHox gene expression (A) and activity (B) was analyzed in mice treated as described in Fig. 1. Data are shown as mean ± s.d. (ns), not significant; *P<0.05; ***P<0.001 versus control mice; [c], *P*<0.001 versus HFD-fed mice. RLU, relative luminescence units. (C) Phosphorylated Rac1 (Phospho-RAC1) was measured by western blot using a specific antibody. (D) Phosphorylated p47^phox^ (Phospho-p47*^phox^*) was analyzed after immunoprecipitating (IP) proteins with anti-p47*^phox^* antibody and western blotting (WB) proteins with anti-p47*^phox^* and anti-phosphoserine. MW, molecular weight.

Because nicotinamide adenine dinucleotide phosphate oxidase (NADPHox; also known as Nox), among others, is capable of causing oxidative stress (De Minicis et al., 2006), we measured activity of this enzymatic complex and gene expression of its components in the liver of control and HFD-fed mice treated with saline, MLT, uric acid or MnTBAP. As Fig. 6A,B show, NADPHox activity and gene expression of *p22^phox^*, *NOX2*, *NOX4*, *RAC1* and *p47^phox^*, five components of the NADPHox complex, were significantly increased in mice on HFD. Moreover, levels of phosphorylated RAC1 (Fig. 6C) and p47*^phox^* (Fig. 6D) were markedly increased in HFD-fed mice. However, treatment of mice with MLT, MnTBAP or uric acid prevented all the changes induced by the HFD.

## DISCUSSION

As expected, mice fed a HFD developed several features of metabolic syndrome and the liver of these animals exhibited a number of features of the NASH phenotype (supplementary material Fig. S2), including increased gene expression of markers of inflammation, fibrosis and apoptosis (Fig. 1A). These lesions, including steatosis, did not appear in animals on the same diet and treated with uric acid, a scavenger of peroxynitrite (Kuzkaya et al., 2005), MLT or MnTBAP, two antioxidants, supporting the notion that nitro-oxidative stress might play a role in the pathogenesis of these lesions.

In the present study, we show that the levels of FFAs and gene expression of proinflammatory cytokines (Fig. 1, Table 1) were significantly increased in the liver of mice on a HFD, which could be responsible for the inflammatory changes found in the liver of these animals. A number of authors have demonstrated the proinflammatory properties of FFAs, particularly of the saturated ones (Han et al., 2010).

Our study also shows evidence of endoplasmic reticulum stress in the liver of HFD-fed mice, which might also be attributable to the increased levels of FFAs in the liver (Wei et al., 2006). This stress can induce apoptosis via CHOP, a transcription factor whose expression is increased in the liver of HFD-fed mice and causes apoptosis (Thorp et al., 2009) (Fig. 1C). Moreover, activation of JNK (Fig. 1C,D), a kinase that can be activated by ROS and blocked by antioxidants (Seki el al., 2012), could also contribute to cell death (Liu et al., 2002). The decrease in steatosis might also be ascribed to the nitro-oxidative stress, because *in vitro* exposition of hepatic proteins to peroxynitrite reduces the amount of ApoB100 and ApoB45 (Solís-Muñoz et al., 2011), two proteins involved in both the assembly of triglycerides into very-low-density lipoprotein (VLDL) and VLDL secretion (Goldberg and Ginsberg, 2006). This effect was avoided in the presence of MLT (Solís-Muñoz et al., 2011). Other authors have also demonstrated that oxidative stress can disrupt ApoB100 structure and reduce its secretion by HepG2 cells (Stewart et al., 2009).

Measurement of OXPHOS complex activity and ATP content in the liver of mice on a HFD demonstrated that this activity was markedly reduced as compared with control mice (Fig. 3). These results are in line with those we have reported in individuals with NASH (García-Ruiz et al., 2006) and in *ob/ob* mice (García-Ruiz et al., 2010; Solís-Muñoz et al., 2011).

Not many studies have been published on the effect of a HFD on hepatic OXPHOS activity (Mingorance et al., 2012), and even fewer on the mechanisms by which these effects are produced. In the present study, we provide evidence supporting that nitro-oxidative stress plays a major role in the pathogenesis of mitochondrial dysfunction provoked by a HFD in mice. Thus, we show that a HFD upregulated iNOS protein expression and caused 3-tyrosine nitration of hepatic and mitochondrial proteins, including OXPHOS subunits (Fig. 2). Likewise, markers of oxidative stress, such as TBARS and 4-hydroxynonenal-protein adducts, were increased and GSH was decreased in the liver of HFD-fed mice (Fig. 1B). Moreover, treatment of mice on a HFD with antioxidants, such as MnTBAP or MLT, or with antiperoxynitrite, such as uric acid, not only prevented nitro-oxidative stress but also increased activity of the OXPHOS complexes (Fig. 3A).

The present study also provides an explanation for the low activity of OXPHOS enzyme complexes in mice on a HFD, because fully assembled complexes and the amount of their subunits, particularly of the mtDNA-encoded subunits (Fig. 4), were markedly decreased in liver mitochondria of these animals. This decrease might be due to a reduced synthesis of OXPHOS subunits, to a defect in their assembly and/or stability, to an increased degradation, or to a combination of all of these mechanisms. The present study shows that gene expression of mtDNA-encoded subunits, but not nDNA-encoded ones, was significantly decreased in HFD-fed mice (Fig. 5A,B). This difference between nDNA- and mtDNA-encoded subunits might be caused by the nitro-oxidative damage of the mtDNA, because 8-OHdG levels, a reliable marker of oxidative DNA damage, were significantly increased in the mtDNA but not in the nDNA (Fig. 5C). Moreover, treatment of HFD-fed mice with antioxidants or with an antiperoxynitrite normalized or even increased gene expression of mtDNA-encoded subunits (Fig. 5B). Although there is little information on this topic, these results are in line with those we found in *ob/ob* mice (García-Ruiz et al., 2006; García-Ruiz et al., 2010; Solís-Muñoz et al., 2011). The role of peroxynitrite is supported by the fact that exposing cells to this anion causes a decline in mtRNA transcripts (Ballinger et al., 2000). Accumulation of mtDNA lesions decreases the synthesis of mtDNA-encoded OXPHOS subunits (Suliman et al., 2002). The reduced synthesis of the mtDNA-encoded subunits can explain the reduced amount of these subunits in mice on a HFD, but not the decrease observed in nDNA-encoded subunits, indicating that other factors might also be implicated.

The decrease in OXPHOS subunits found in HFD-fed mice might also contribute to its increased degradation. This mechanism could explain not only the low amount of mtDNA-encoded subunits found in HFD-fed mice, but also the reduction in subunits encoded by the nDNA, whose synthesis remains normal. The fact that mtDNA-encoded polypeptides are decreased significantly more than nDNA-encoded subunits might be attributed to the combination of both mechanisms – the low synthesis and the enhanced degradation of these subunits. Nitration of mitochondrial proteins might explain their degradation (Murray et al., 2003). In a previous study, we showed that *in vitro* incubation of normal mitochondrial proteins with peroxynitrite induced the degradation of the OXPHOS subunits (García-Ruiz et al., 2010). In these experimental conditions, defects in the synthesis or in the assembly of mitochondrial complexes can be excluded. In the present study, we show that OXPHOS complexes and their subunits were intensely 3-tyrosine nitrated (Fig. 2) and that treatment of mice on a HFD with uric acid decreased liver tissue nitration (Fig. 2A) and normalized the amount of fully assembled OXPHOS complexes, mitochondrial subunits (Fig. 4) and enzymatic activity of the OXPHOX complexes (Fig. 3A). Peroxynitrite is produced by the reaction of nitric oxide with superoxide anion and there is evidence showing that nitric oxide and superoxide anion formation is increased in the liver of individuals with NASH and *ob/ob* mice. Thus, Sanyal et al. found that there was considerable staining for 3-NT in individuals with fatty liver or NASH (Sanyal et al., 2001), Laurent et al. showed that the concentrations of nitrites and nitrates were significantly increased in liver homogenates of *ob/ob* mice (Laurent et al., 2004), and we demonstrated that iNOS protein expression is upregulated in the liver of *ob/ob* mice (García-Ruiz et al., 2006). In the present study, we show that liver tissue and mitochondrial proteins were intensely tyrosine nitrated in HFD-fed mice (Fig. 3B).

An additional finding in our study was that a HFD also increased the amount of UCP-2 protein in mice (Fig. 1C), which could contribute to the decrease in both ATP formation (Fisler and Warden, 2006) and ROS generation. That is, the elevated UCP-2 expression might be a protective mechanism against oxidative stress. ROS (Echtay et al., 2002) and FFAs (Chan et al., 2004), among others, are factors involved in the upregulation of *UCP-2* gene expression.

The cause of the oxidative stress leading to the dysfunction of OXPHOX remains unclear and requires further studies. Potential sources of oxidative stress are cytochrome P4502E1 (Emery et al., 2003), xanthine oxidase (Nanduri et al., 2013), NADPHox (De Minicis et al., 2006) and liver mitochondria (Sanyal et al., 2001). Our study demonstrates that mice on a HFD have elevated NADPHox activity and gene expression of its components as well as phosphorylation of p47*^phox^* and RAC1, two cytosolic components of the NADPHox complex (Fig. 6). This oxidase is a multiprotein complex capable of causing oxidative stress (De Minicis et al., 2006). Activity of NADPHox can be induced by FFAs (Hatanaka et al., 2013), TNFα (Mohammed et al., 2013) and TGFβ1, all of which are increased in the liver of obese mice. The latter growth factor is known to induce gene expression of *NOX4*, a member of NADPHox (Bai et al., 2014). Therefore, we could speculate that these agents increase gene expression and enzyme activity of components of the NADPHox complex, leading to oxidative stress and OXPHOS dysfunction. Because the latter is also an important cellular source of ROS (Fromenty et al., 2004), it could create a vicious cycle that would contribute to increasing the oxidative stress. Although little information exists on the effects of NADPHox on OXPHOS function, Nox4, one member of the NADPHox family located in the mitochondrial inner membrane, has been shown to inhibit activity of complex I of the OXPHOS complexes (Kozieł et al., 2013). However, as mentioned above, the relationship between NADPHox, and other oxidative systems, with OXPHOS dysfunction require further studies. Likewise, new studies are required to determine the role played by FFAs and TNFα in the eventual activation of NADPHox.

We conclude that a HFD decreases OXPHOS enzymatic activity, which can be ascribed to a decreased amount of fully assembled complexes. This defect is due to reduced synthesis and likely to increased degradation of their subunits. Antioxidants and antiperoxynitrite prevent all these changes, suggesting that nitro-oxidative stress plays a key role in the pathogenesis of these alterations. Treatment with these agents might be useful in preventing NASH in humans.

## MATERIALS AND METHODS

### Animal model of NAFLD

All procedures were carried out in accordance with the Spanish Guidelines for the Care and Use of Laboratory Animals. The 6-week-old male C57BL/6J mice were purchased from Charles River Laboratory (Charles River Laboratories España SA, Santa Perpetua de la Mogoda, Spain). Animals were housed at constant room temperature (23°C) under 12-hour light/dark cycles with *ad libitum* access to water and laboratory diet. Thirty mice were distributed in five groups: (1) control, included six C57BL/6J mice fed a standard chow diet and treated with 200 μl 0.8% saline solution; (2) group HFD contained six mice on a HFD (Harlan Laboratories, Madison, WI) consisting of 21.2% (42% kcal) fat, 17.3% (15.2% kcal) protein and 35% (42.7% kcal) carbohydrate and treated with 200 μl 0.8% saline solution; (3) group MLT was composed of six mice on a HFD treated with 10 mg/kg body weight/day MLT (Sigma-Aldrich Química SA, Tres Cantos, Spain); (4) group MnTBAP consisted of six HFD-fed mice treated with 10 mg/kg body weight/day MnTBAP (Calbiochem, San Diego, CA), a mimic of manganese superoxide dismutase; (5) group UA was made up of six HFD-fed mice treated with 20 mg/day uric acid. Uric acid (Sigma-Aldrich Química SA, Tres Cantos, Spain) was used as a suspension of 20 mg in 200 μl 0.8% saline solution. Saline, MLT and MnTBAP solutions and uric acid suspension were administered intraperitoneally (i.p.). Diets and intraperitoneal treatments were maintained for 28 weeks. Body weight and food intake were measured every 2 weeks. Food intake was calculated by regularly weighing the amount of food given to mice and calculating the daily loss. Food but not water was withdrawn overnight before mice were killed. Animals were anesthetized and killed at 34 weeks of age, and the liver was rapidly harvested for further analysis. A portion of the liver tissue was placed in a 10% formaldehyde solution and routinely processed for histological assessment. Sections were stained with hematoxylin-eosin and Masson trichrome. Nitration of proteins by peroxynitrite [3-nitrotyrosine (3-NT)] in the liver was assessed as described elsewhere (García-Ruiz et al., 2006). Plasma glucose, triglycerides and aminotransferases were measured using a conventional automatic analyzer. Plasma FFA levels were determined using the ‘Free Fatty Acids, Half Micro Test’ kit (Roche Diagnostics GmbH, Penzberg, Germany).

### Quantitative real-time polymerase chain reaction

Quantitative real-time polymerase chain reaction was performed following the method described elsewhere (García-Ruiz et al., 2010). The sequence of primers used in these experiments is shown in supplementary material Table S1. Expression of complex-I subunits was normalized to that of β-actin.

### Extraction of hepatic and mitochondrial proteins, and western blot analysis

Total protein was extracted from liver tissue using a standard protocol in our laboratory (Santiago et al., 2004). Liver mitochondria were isolated from liver homogenates by differential centrifugation as described by Turko et al. (Turko et al., 2001). Proteins were separated and transferred to an Immobilon membrane (Millipore, Bedford, MA) as previously described (Solís-Herruzo et al., 1999). After electrotransfer, the filters were incubated with appropriate polyclonal antibody against 3-NT (Upstate Biotechnology, Lake Placid, NY), iNOS, CHOP, UCP-2, β-actin, PPARγ, adiponectin, p47*^phox^*, phosphorylated Rac1 (Santa Cruz Biotechnology, Santa Cruz, CA) and phosphorylated serine (Sigma-Aldrich, Alcobendas, Spain). Signals were detected using the ECL Western Blotting Detection Reagent (Amersham Ibérica, Madrid, Spain).

### Determination of aldehyde-protein adducts

This was performed using the ‘OxiSelect™ HNE Adduct ELISA Kit’ (Cell Biolabs Inc., San Diego, CA) according to the manufacturer’s protocol.

### Determination of lipid peroxidation and glutathione content in mitochondria

Lipid peroxidation was determined by measuring TBARS in liver homogenate as described by Ohkawa et al. (Ohkawa et al., 1979). Mitochondrial glutathione (GSH) was measured using the Eady et al. modification of the Tietze’s assay (Eady et al., 1995).

### Adiponectin

Level of adiponectin was determined in mouse plasma using an ELISA assay (Invitrogen, Life Technology, Frederick, MD).

### OXPHOS enzyme activity assays

Enzyme activity of the OXPHOS complexes was measured in frozen liver tissue as described elsewhere (García-Ruiz et al., 2006), expressed as nanomoles of substrate used per minute per milligram of protein and, to correct for the hepatic content of mitochondria, referred to as a percentage of the specific activity of citrate synthase (CS).

### Assessment of fully assembled OXPHOS complexes

Mitochondrial proteins were extracted and separated as described elsewhere (García-Ruiz et al., 2010). Enhanced chemiluminescence (ECL) signals were quantified using the ImageJ image analysis software (Rasband, 2012).

### In-gel activity assays

OXPHOS complexes were separated by one-dimensional BN-PAGE as described previously (García-Ruiz et al., 2010) and complex-I in-gel activity was measured as described by Nijtmans et al. (Nijtmans et al., 2002).

### Second-dimension electrophoresis for assessing complex subunits

For second dimension BN/SDS-PAGE, we followed the procedure described elsewhere (Soung et al., 2004). Western blotting was performed using primary antibodies against subunits NDUFA9, NDUFS3, NDUFB6, NDUFA6, NDUFV1, NDUFV2, MTND1, MTND6 and MTND4L (complex I); SDHA (complex II); UQCRC2, UQCRFS1 and MTCYTB (complex III); COX4 and MTCO1 (complex IV); and ATP5A1 and MTATP8 (ATP synthase) (Molecular Probes Inc., Eugene, OR). Antibody against MTND1, MTND6 and MTND4L were obtained from Santa Cruz Biotechnology Inc. (Santa Cruz, CA).

### Measurement of total ATP content and ATP:ADP ratio in mouse liver

Liver samples were homogenized in perchloric acid and centrifuged at 15,000 ***g*** for 2 minutes. Supernatants were collected and 30 μl was added to a 96-well plate and then brought up to 50 μl with ATP assay buffer. ATP reaction mix and ATP measurement was performed using the ATP Colorimetric/Fluorometric Assay Kit (BioVision Research Products, Milpitas, CA) according to the manufacturer’s protocol. The ADP:ATP ratio was measured by luminometry using the commercial assay kit ApoSENSOR™ ADP/ATP Ratio Assay Kit (BioVision Research Products, Mountain View, CA).

### Measurement of 8-OHdG in nDNA and mtDNA

Oxidative damage to nDNA and mtDNA was determined following the procedure described elsewhere (García-Ruiz et al., 2010).

### Immunoprecipitation

The immunoprecipitation assays were performed as previously described (Lang et al., 2000). Liver proteins were precipitated with polyclonal antibodies against p47*^phox^* (Santa Cruz Biotechnology, Santa Cruz, CA). Immunocomplexes were recognized by western blot using specific antibodies against p47*^phox^* and anti-phosphoserine (Sigma-Aldrich).

### NADPHox activity

NADPHox activity was measured following the procedure described by Jalil et al. (Jalil et al., 2005).

### c-Jun N-terminal kinase

Level of JNK was measured using the commercial assay kit KinaseSTAR™ JNK Activity Assay Kit (BioVision Research Products, Milpitas, CA) according to the manufacturer’s protocol.

### Statistical analysis

These analyses were carried out using the SPSS Statistical Software for Windows, version 9 (SPSS Inc., Chicago, IL). The unpaired *t*-test was used to assess the significance of differences between means. All results were expressed as mean ± s.d. *P*-values <0.05 were considered significant.

## Supplementary Material

Supplementary Material
