# Supplementary material for: High-fat diet decreases activity of the oxidative phosphorylation complexes and causes nonalcoholic steatohepatitis in mice
Source: Dis Model Mech. 2014 Sep 26;7(11):1287–96. doi: 10.1242/dmm.016766 (PMC4213732; doi:10.1242/dmm.016766)
Supplement: Supplementary Material [file supp_7_11_1287__index.html]

High-fat diet decreases activity of the oxidative phosphorylation complexes and causes nonalcoholic steatohepatitis in mice — Supplementary Material 

# High-fat diet decreases activity of the oxidative phosphorylation complexes and causes nonalcoholic steatohepatitis in mice

## DMM016766 Supplementary Material

**Files in this Data Supplement:**

- **Supplementary Material**
