# Supplementary material for: High-fat diet decreases activity of the oxidative phosphorylation complexes and causes nonalcoholic steatohepatitis in mice
Source: Dis Model Mech. 2014 Sep 26;7(11):1287–96. doi: 10.1242/dmm.016766 (PMC4213732; doi:10.1242/dmm.016766)
Supplement: Supplementary Material [file supp_7.11.1287_DMM016766.pdf]

Figure S1.

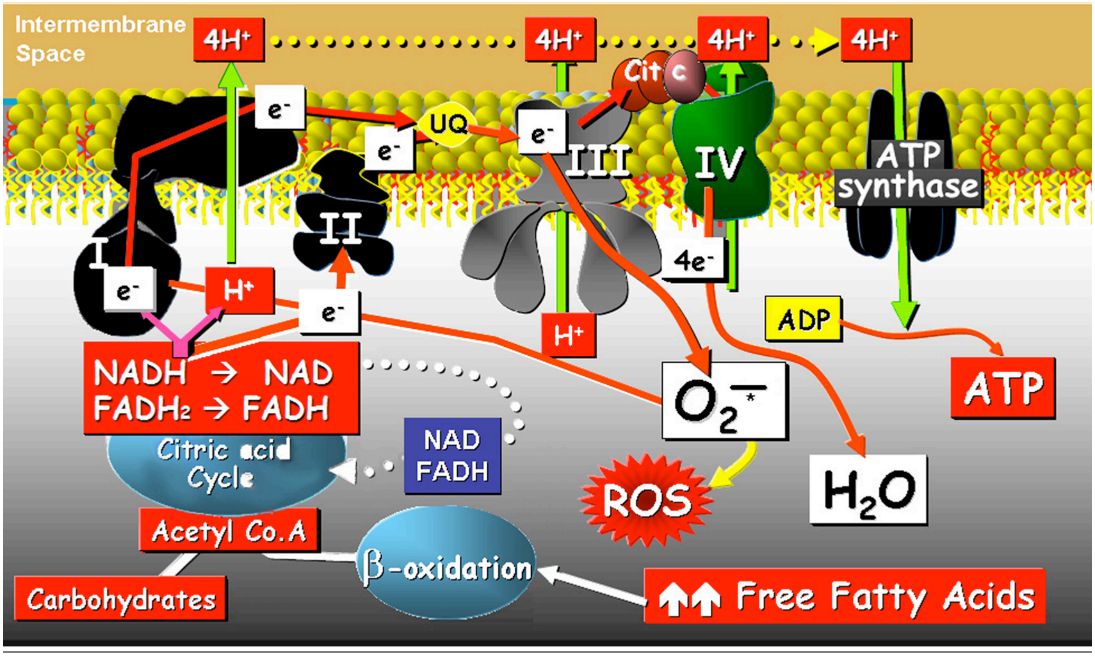

Oxidative phosphorylation and mitochondrial respiratory chain. Free fatty acids (FFAs) are β-oxidized in the mitochondria leading to the formation of acetyl-CoA and the conversion of oxidized nicotinamide adenine dinucleotide (NAD) and flavin adenine dinucleotide (FADH) into NADH and FADH2. Afterward, both dinucleotides are reoxidized into NAD and FADH, respectively, by the MRC. This chain consists of four enzymatic complexes (complexes I to IV) and two intermediary substrates (ubiquinone (UQ) and cytochrome c). Complex I (NADH dehydrogenase) accepts electrons from NADH and transfers them to UQ, which also receives electrons from complex II (succinate dehydrogenase). UQ passes these electrons to complex III (cytochrome bc_1 complex), which transfers them to cytochrome c. Electrons from this carrier are removed and finally combined with oxygen and protons to form water. This transfer of electrons along the MRC is coupled with the exit of protons from the mitochondrial matrix into the intermembrane space creating an electrochemical potential across the inner membrane. Protons return to the matrix through the ATP synthase, causing the conversion of ADP into ATP. In normal conditions, a small fraction of electrons (5%) flowing along the MRC reacts with oxygen to form ROS. However, the mitochondrial ROS production increases markedly when the electron flow along the MRC is impaired. Coupling of the electron transfer along the MRC with the exit of protons is a key step for ATP production. In some conditions, these two processes are uncoupled and protons flow back directly from the intermembrane space across the inner mitochondrial membrane into the mitochondrial matrix dissipating the potential energy in thermogenesis rather than in ATP production. Uncoupling proteins favor this flow of protons back to the matrix decreasing ATP and ROS production.

Figure S2

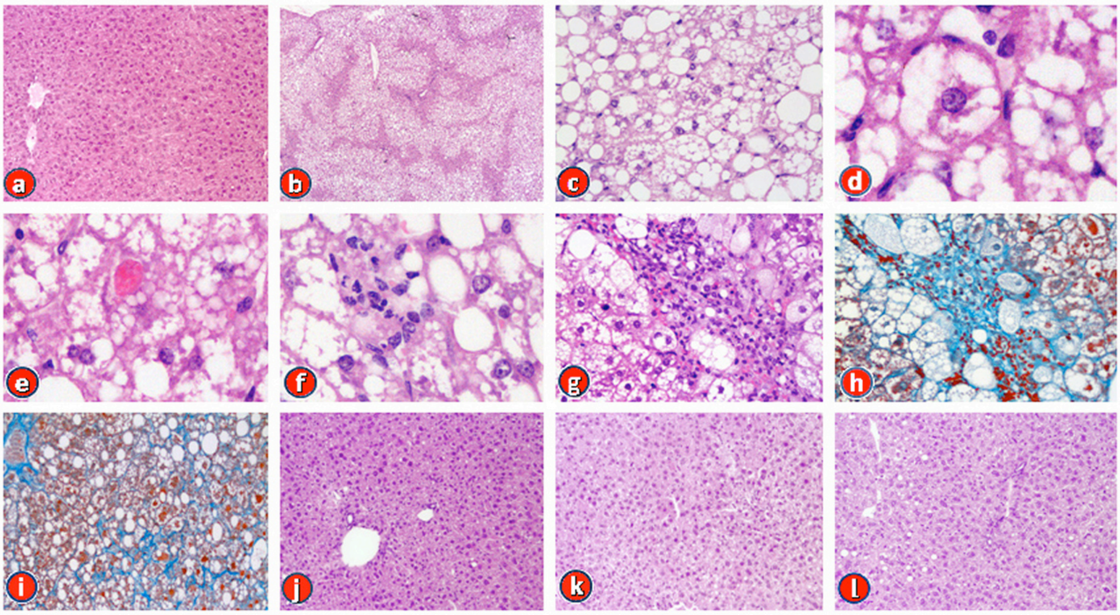

Liver histology. (**a**) Mouse on standard chow diet and treated with saline i.p. for 28 weeks. (**b-i**) Mice on HFD treated with saline i.p. for 28 weeks. (**b**) Severe steatosis. (**c**) Macro- and microvesicular steatosis. (**d**) Hepatocyte with ballooning degeneration and Mallory bodies. (**e**) Eosinophilic body. (**f**) Mixed neutrophilic-lymphocytic infiltrate. (**g**) Inflammatory infiltrate. (**h**) Fibrosis. (**i**) Perisinusoidal fibrosis. (**j**) Mouse on HFD treated with melatonine i.p. for 28 weeks. (**k**) Mouse on HFD treated with uric acid i.p. for 28 weeks. (**l**) Mouse on HFD treated with MnTBAP i.p. for 28 weeks. Liver samples were stained with hematoxylin-eosin (**a-g, j-l**) or Masson's-trichrome stain (**h,i**). Magnification 25x (**b**), 100x (**i**), 200x (**a,c,e,f,g,h,j,k,l**), 400x (**d**).

Table S1.

Primers used in quantitative real-time polymerase chain reaction

Primer name	Direction	Sequence
Mouse NDUFA9	Sense	5′-CAT TAC TGC AGA GCC ACT-3′
	Antisense	5′-ATC AGA CGA AGG TGC ATG AT-3′
Mouse NDUFB6	Sense	5′-ATA ACT TTT TGC GGG ACG GG-3′
	Antisense	5′-CAG GAA AAT CTC TCA TTG GTG-3′
Mouse NDUFS3	Sense	5′-AGG AAC ATG GCG GCG GCT GC-3′
	Antisense	5′-ATT TCA GCC ACA TAC TCT CC-3′
Mouse MTND1 (ND1)	Sense	5′-TGC ACC TAC CCT ATC ACT C-3′
	Antisense	5′-ATT GTT TGG GCT ACG GCT C-3′
Mouse MTND2 (ND2)	Sense	5′-ATG AGT AGG CCT GGA ATT C-3′
	Antisense	5′-ATC AGA AGT GGA ATG GGG C-3′
Mouse MTND4 (ND4)	Sense	5′-ATA ATT ATA ACT AGC TCA ATC TGC-3′
	Antisense	5′-TCG TAG TTG GAG TTT GCT AG-3
Mouse MTND4L (ND4L)	Sense	5′-CTC ACC ATA GCC TTC TCA C-3′
	Antisense	5′-CGT AAT CTG TTC CGT ACG TG-3′
Mouse MTND6	Sense	5′-TGT ATG AGG TTG ATG ATG TTG G-3′
	Antisense	5′-CCG CAA ACA AAG ATC ACC C-3′
Mouse β-actin	Sense	5′-ATG GAT GAC GAT ATC GCT G-3′
	Antisense	5′-GTT GGT AAC AAT GCC ATG TTC-3′
Mouse UQCRC1 (core 1)	Sense	5′-CCT ACA GCA CTC GAG AGC AC-3′
	Antisense	5′-AGG TGT GCC CTG GAA TGC TG-3′
Mouse UQCRC2 (core 2)	Sense	5′-TCC CTC AAA GTT GCC CC-3′
	Antisense	5′-GCA AGA CGT AGT AAA TGT GAG-3′
Mouse UQCRFS (FeS)	Sense	5′-GAT GTC AAG GTG CCC GAC TT-3′
	Antisense	5′-GAT CTC GAT CTT CGA CAT GG-3′
Mouse MT CYB	Sense	5′-CTT TGG GTC CCT TCT AGG AGT CTG-3′
	Antisense	5′-GCT GTG GCT ATG ACT GCG AAC AG-3′
Mouse SDHA (70 kDa)	Sense	5′-CAT ACT GTT GCA GCA GCA CAG G-3′
	Antisense	5′-CCA CCA AAT GCA CGC TGA TA-3′
Mouse COX4 (COX IV)	Sense	5′-GAG CAC ATG GGA GTG TTG TG-3′
	Antisense	5′-CTG TCT TCC ATT CAT TGG TGC C-3′
Mouse MTCOX1	Sense	5′-GCT GAA GGA GAA GGA GAA G-3′
	Antisense	5′-ATA CAC ATA GCT CTT CTC CC-3′
Mouse ATP5	Sense	5′-GGT CAT CCT TTG TTG GTG C-3′
	Antisense	5′-GAG AAT TCC ACC ATC CCT TC-3′
Mouse MTATP6	Sense	5′-CCA CAC ACC AAA AGG ACG AAC ATG A-3′
	Antisense	5′-CGG ACT GCT AAT GCC ATT GGT TG-3′
Mouse TNFα	Sense	5′-GAG GCA CTC CCC CAA AAG-3′
	Antisense	5′-GGG TCT GGG CCA TAG AAC TG-3′
Mouse IFNγ	Sense	5′-CTC AAG TGG CAT AGA TGT GG-3′
	Antisense	5′-CAG GTG TGA TTC AAT GAC GC-3′
Mouse MCP-1	Sense	5′-CCA CGT GTT GGC TCA GC-3′
	Antisense	5′-AGC ACA GAC CTC TCT CTT G-3′
Mouse RCP	Sense	5′-GAC ACT CAG CCC CAA TGT TTT G-3′
	Antisense	5′-GTC CTC TAG TGC TGA GGA C-3′
Mouse Caspase 3	Sense	5′-GGA TGT GGA CGC AGC CAA-3′
	Antisense	5′-CCT TCA TCA CCA TGG CTT AG-3′
Mouse Collagen 1α(I)	Sense	5′-CTT CCC TGG TGC AGT TGG T-3′
	Antisense	5′-GCA ATA CCA GGA GCA CCA TTG-3′
Mouse TGFβ	Sense	5′-CAG AGC TGC GCT TGC AGA GAT TAA-3′
	Antisense	5′-CAG TGA GCG CTG AAT CGA AAG C-3′
Mouse NOX2	Sense	5′-GCA GCC TGC CTG AAT TTC AAC T-3′
	Antisense	5′-AGA GAG AGC TAT TGA ATA CCG G-3′
Mouse NOX4	Sense	5′-CCA AAT GTT GGG CCT AGG ATT G-3′
	Antisense	5′-CAG TTC AAG GGAA ATC TTC ACT GTA G-3′
Mouse p47^{phox}	Sense	5′-GCC TGA GAC ATA CCT GGT GCC CAA A-3′

	Antisense	5′-GAC CCA ACC TCG CTT TGT CTT CAT-3′
Mouse p22*phox*	Sense	5′-GGA CGT TTC ACA CAG TGG TAT TTC G-3′
	Antisense	5′-CAC CTC GGA TGG CTG CCA GCA G-3′
Mouse Rac1	Sense	5′-CAT CCC CAC CGT CTT TGA CAA-3′
	Antisense	5′-ACG AGG ATG ATA GGA GTA TTG GG-3′
Mouse 18S rRNA	Sense	5′-GGA TAC CGC AGC TAG GA-3′
	Antisense	5′-TCA TGG GAA TAA CGC CG-3′
